# Importance of Muscle Power Variables in Repeated and Single Sprint Performance in Soccer Players

**DOI:** 10.2478/hukin-2014-0022

**Published:** 2014-04-09

**Authors:** Manuel López-Segovia, Alexandre Dellal, Karim Chamari, Juan José González-Badillo

**Affiliations:** 1 Research Section, Murcia Soccer Federation, Murcia, Spain.; 2 INNOVA Health & Sport Institute, Murcia, Spain.; 3 Tunisian Research Laboratory “Sport Performance Optimisation”-National Center of Medicine and Science in Sport (CNMSS)El Menzah, Tunisia.; 4 Santy Orthopedicae clinical, sport science and research department Lyon, France.; 5 Research Laboratory “Sport Performance Optimisation”, National Center of Medicine and Science in Sport.; 6 Department of Exercise Science, University of Pablo de Olavide, Seville, Spain.

**Keywords:** repeated-sprint ability, strength, testing, soccer, velocity

## Abstract

This study examined the relationship between lower body power and repeated as well as single sprint performance in soccer players. The performance of nineteen male soccer players was examined. The first testing session included the countermovement jump (CMJL) and the progressive full squat (FSL), both with external loads. Power in the CMJL and FSL was measured with each load that was lifted. The second session included a protocol of 40-m repeated sprints with a long recovery period (2 min). The number of sprints executed until there was a 3% decrease in performance for the best 40-m sprint time was recorded as a repeated sprint index (RSI). The RSI was moderately associated with power output relative to body mass in the CMJL and FSL (r = 0.53/0.54, p ≤ 0.05). The most and least powerful players (determined by FSL) showed significant differences in the RSI (9.1 ± 4.2 vs. 6.5 ± 1.6) and 10 m sprint time (p ± 0.01). Repeated and single sprints are associated with relatively lower body power in soccer players.

## Introduction

Previous studies have examined physical performance, especially high-intensity activities in a competitive soccer match (Bradley et al., 2009;Di Salvo et al., 2010). Although these analyses were influenced by variables such as game location (Di Salvo et al., 2010; Impellizzeri et al., 2008) and a player’s sports level (Impellizzeri et al., 2008; Rampinini et al., 2009), the ability of soccer players to repeat high-intensity actions is considered to be a key factor in elite soccer (Rampinini et al., 2009). For this reason, different researchers have attempted to clarify the application of various repeated sprint ability (RSA) protocols (Girard et al., 2011).

In a sport like soccer, which is characterized by intermittent efforts, aerobic capacity is an essential ability (Hoff, 2005;Rampinini et al., 2009), and it has been shown that it could influence RSA (Glaister, 2005). Moreover, the application of strength is also an essential component of soccer players’ fitness to execute the constant muscular adjustments necessary for different actions (Hoff, 2005). Indeed, the power output of the lower limbs as the product of strength and velocity has been associated with soccer players’ sprint performance (López-Segovia et al., 2011). In this context, several studies have examined the possible associations between sprint ability and various strength and power measures in different exercises (López-Segovia et al., 2011; Wisloff et al., 2004). However, the relationships between RSA and power measurement that are obtained in isoinertial exercises, which are widely used in soccer players’ strength training (e.g. squats and squats jumps) (López-Segovia et al., 2010; Wong et al., 2010), remain unknown. Accordingly, it has been shown that power training could be used for youth soccer players, inducing improvement in sprint performance without impacting endurance (Wong et al., 2010).

To analyze the relationship between fitness variables and RSA, the majority of protocols have used short-duration sprints (< 10 s) interspersed with short recovery periods (< 60 s) (Girard et al., 2011). However, elite players perform a high-intensity run (> 19.8 km/h) every 72 to ∼90 seconds (Bradley et al., 2009) and in an analysis that focused on very high-intensity activities (> 25 km/h) elite soccer players performed from 17 to 30 sprints during an official match depending on their playing positions (Di Salvo et al., 2010). In this context, it was shown by Faude et al. (2012) that straight sprinting was the most frequent action in soccer goal situations, stressing the importance of power and speed abilities in decisive situations in professional soccer.

However, little attention has been given to RSA interspaced by long recovery periods. Moreover, different protocols have been performed with a determined number of sprints for all subjects without taking into account the individual player’s capacity to endure RSA nor their positional roles. This issue is frequently ignored by practitioners and researchers who design the same number of sets, repeated sprints, or interval training without considering the individual capacities of each player (Buchheit et al., 2010).

In this regard, the first aim of the present study was to examine soccer players’ individual performance loss with an unequal number of sprints and to analyse if this decrease was associated with lower limb power.

The second aim was to determine the importance of lower body power in repeated and single sprints in soccer players. It was hypothesized that power output is significantly related to single and repeated sprint ability and that it has a very important role with respect to individualized repeated sprint training in soccer players.

## Material and Methods

### Participants

Nineteen semi-professional (Spanish Third Division) outfield male soccer players participated in the study (mean ± *SD*: age 21.2 ± 2.1 y, body mass 75.6 ± 6.8 kg, and body height 178 ± 0.1cm). The players trained four times per week (between 90 to 120 min) and played a weekly match. The present investigation was approved by the Research Ethics Committee of Pablo de Olavide University, Seville, and was conducted in accordance with the Declaration of Helsinki. The subjects received information about the characteristics and procedures of the study, their voluntary participation in it, the possibility of withdrawing at any moment without penalty, and the confidentiality of the data.

### Measures

The present study used a cross-sectional experimental design to examine the relationship between mechanical power output variables and RSA with long recovery periods in semi-professional soccer players. All testing procedures were completed at the end of a twice weekly resistance-training period performed during the mid-season. The programme included full squats with loads close to the relative load that maximized mechanical power output (Sánchez-Medina et al., 2010) in the full squat (FSMax_MP_) (2–3 sets of 4–6 repetitions) and countermovement jumps with an external load of 25% of the FSMax_MP_ (2–3 sets of 3–4 repetitions). Consequently, all players were familiar with all the testing procedures.

To determine the importance of power output measures in RSA and a single sprint, two testing sessions were conducted that were separated by a week and were carried out at least 48 hours after the most recent game. The first testing session included measuring power output under standardized conditions in a laboratory through the following progressive tests: a countermovement jump with an external load (CMJ_L_) and a full squat with an external load (FS_L_). The second session included a 40 m sprint protocol in which players repeatedly sprinted until there was a 3% decrease in performance (2 min of passive recovery separated sprint trials). All players were verbally encouraged to give their maximum effort.

### Procedures

#### Lower limb power test

Lower limb explosive power was measured in standardized laboratory conditions with the following progressive external-load tests on the Smith machine (Fitness Line, Peroga, Spain) ensuring displacement: coutermovement jump (CMJ_L_) and full squat (FS_L_) (López-Segovia et al., 2010).

To measure the power of the CMJ and FS with external loads, the bar of the Smith machine was equipped with a linear velocity transducer. This dynamic measurement system (T-Force System, Ergotech, Murcia, Spain) automatically calculates the peak and mean power of every repetition without adding the body mass of each player to the loaded bar; it provides auditory velocity and displacement feedback, and stores the data for analysis. This system consists of a linear velocity transducer interfaced with a personal computer by means of a 14-bit resolution analogue-to-digital data acquisition board and custom software. Instantaneous velocity was sampled at a frequency of 1000 Hz and subsequently smoothed with a fourth-order low-pass Butterworth filter with a cut-off frequency of 10 Hz. The validity and reliability of this system has been previously documented (Sánchez-Medina et al., 2010).

The power output of the jump was measured after two CMJ with each of the following external loads: 20 kg, 30 kg, and 40 kg (López-Segovia et al., 2010). The subjects performed a 15 min standardized warm-up consisting of a low intensity run, several accelerations, and 15–20 interspersed full squats followed by five CMJ. Both exercises were performed without an external load. The players were instructed to keep their hands on the bar of the Smith machine during each jump. The best score for peak power during the concentric phase of the repetitions performed with each external load (PPCMJ_L_), and the sum of the maximum peak power output with 20 kg, 30 kg, and 40 kg (PPCMJ_20-30-40_) was recorded for analysis. Four minutes of recovery was given between each jump. The coefficient of variation (CV) and the intra-class correlation coefficient (ICC) for this test was reported as 4.0–4.3% and 0.97–0.93, respectively (López-Segovia et al., 2010).

Five minutes after the CMJ test with an external load, the power output and speed in the concentric phase of the full squat for each load used was measured (CV 2.9–4%, ICC 0.92–0.94; López-Segovia et al., 2010). The test began with a resistance of 20 kg, with increments of 10 kg and with 4 min of recovery between each series of repetitions. The number of repetitions performed by each player with each load was determined according to the speed of the first repetition (López-Segovia et al., 2010). Three repetitions were performed with loads in which the subject moved the bar with an average speed of ≥ 1m·s^−1^ during the concentric phase. When the subject moved the bar slower (i.e. < 1m·s^−1^), only two repetitions were performed (López-Segovia et al., 2010). The players were instructed to keep their hands on the bar of the Smith machine during each repetition. Then, from an upright position each player descended slowly until they felt the contact between their posterior thighs and their shanks, then immediately ascended at maximal velocity to the upright position. The best data for mean power output during the concentric phase of the repetitions performed with each external load was utilized for analysis (FS_MP_). The test ended for each subject when the power output for the last load lifted was lower than that of the previous load (López-Segovia et al., 2011). The load that maximized the mechanical mean power output in FS was retained as the player’s maximum mean power load, and the power output registered with this load was retained as player’s maximum mean power output (FSMax_MP_).

#### Repeated Sprint Protocol

After a standardized warm-up consisting of 15 minutes of low intensity running, 3 × 40 m accelerations, and 2 × 40 m sprints at maximal speed with 1 min of rest in-between, the subjects passively rested for 3 minutes. Then, the players ran 40 m sprints with 2 minutes of rest in-between, with instructions to run each 40 m as fast as possible. The 2 × 40 m sprints for the warm-up were not included in the test. Immediately after completion of each sprint, the players walked back to the starting line and waited for the next sprint. Each player continued sprinting until there was a 3% decrease in his 40 m sprint performance. To verify that this degree of fatigue had been attained a second attempt was allowed. In the present experiment, each time the subject showed a 3% decrease in sprint performance, the time of the further additional attempt was always within the range of the fatigued sprint (i.e. sprinting time greater than 3%). The performance reliability of the repeated 40 m sprint times with two minutes of recovery was previously determined (Balsom et al., 1992). The starting position was standardized, with the lead-off foot behind the starting line. Photoelectric cells (Brower Wireless Sprint System, Utah, USA) were placed at 0 m, 10 m, 20 m, and 40 m from the starting line at a height of 40 cm. The number of 40-m sprints that were executed was recorded as the repeated sprint index (RSI). Times at 0–10 m (T_10_), 0–20 m (T_20_) and 0–40 m (T_40_) were recorded. The CVs for these variables were 1.2–2.6%, and the ICCs were 0.92–0.99.

#### Blood lactate evaluation

Blood lactate concentration was measured prior to testing, after the warm up, and just before the repeated sprint test (La_PRE_) by a finger prick using a portable lactate analyser (Accutrend Lactate, Roche Diagnostics, Basel, Switzerland). The reliability and accuracy of this portable analyser have been documented previously (Baldari et al., 2009). Immediately after the last sprint, blood lactate concentration (La_POST_) was measured again.

### Statistical analysis

Descriptive statistics are presented as means ± standard deviation (SD). Pearson correlation coefficients were used to determine the interrelationships between variables. In order to assess the importance of lower body power output for sprint and repeated sprint performance, the players were divided into the most (*n* = 9) and least (*n* = 9) powerful players based on two different variables related to body mass: FSMax_MP_ and CMJ_20–30–40_. Both groups were compared using ANOVA. The following criteria were adopted for interpreting the magnitude of the correlation (*r*) between the measures: ≤ 0.1, trivial; > 0.1–0.3, small; > 0.3–0.5, moderate; > 0.5–0.7; large, > 0.7–0.9; very large; and > 0.9–1.0, almost perfect (Hopkins et al., 2009). The alpha-level was set at p ≤ 0.05.

## Results

Table 1 shows the results of different evaluated variables. When comparing both the single and repeated sprint performance according to the players’ power output data in FS_L_ and CMJ_L_ tests (Table 2), the most powerful players were faster in all variables measured. These differences were statistically significant in T_10_ (*p* ≤ 0.01). In addition, the most powerful players were able to sprint more times (ranging from 6 to 15 sprints) than those who were less powerful in FS_L_ (ranging from 4 to 8 sprints) without a significant loss of performance (*p* ≤ 0.05).

The relationships between mechanical power output variables obtained in the FS_L_ and CMJ_L_ tests and RSI and single sprint performances are presented in Table 3. Significant correlations were obtained between FS_MP_ and best sprint times at 10 m, 20 m, and 40 m that ranged from large to very large (*r* = −0.566/−0.727, *p* ≤ 0.05/0.01). No relationships were found between these variables and the RSI. With regard to the CMJ_L_ test power output results, the correlation of PPCMJ_20–30–40_ per kg body mass with the RSI and all sprints measured was large (*r* = 0.591/−0.640, *p* ≤ 0.05/0.01), and PPCMJ_L_ showed a significant relationship with the majority of sprints measured (*r* = 0.500/−0.663, *p* ≤ 0.05/0.01).

**Figure 1.**
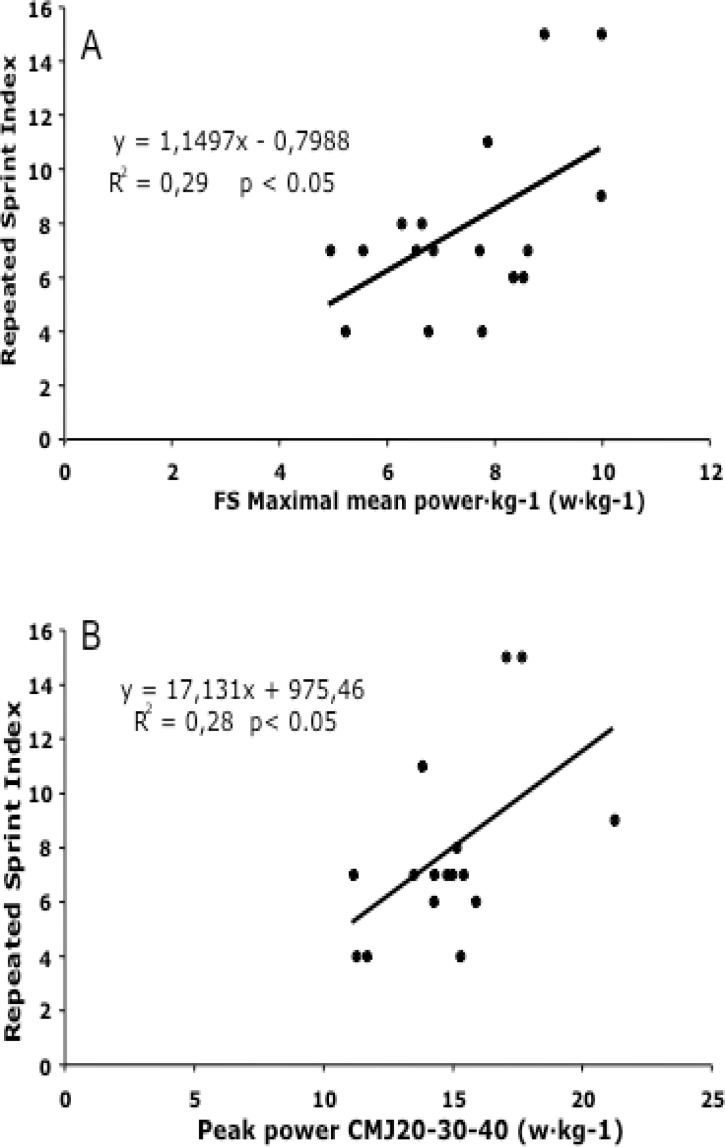
Relationships between repeated sprint index and A) full squat maximal mean power related to body mass, and B) sum of peak power in CMJ with 20, 30 and 40kg by kg of body mass.

No significant correlation was found between the FSMax_MP_ and RSI, but large correlations were obtained between the RSI and both variables related to body mass, the FSMax_MP_·kg^−1^ (*r* = 0.539, *p* ≤ 0.05), and the CMJ_20–30–40_·kg^−1^ (*r* = 0.591, *p* ≤ 0.05) (Figure 1).

## Discussion

The main purpose of this study was to examine the soccer players’ individual performance loss within RSA and to determine if this decrease was associated with lower limb power. The main findings of the present study showed that variables of lower body power expressed relative to body mass (i.e. FSMax_MP_ and PPCMJ_20–30–40_), were related to soccer players’ capacity to maintain maximal speed in a repeated sprint protocol (Figure 1).

In contrast to the present results, previous studies have reported that players obtaining the highest power output in a 6-s cycle (Bishop and Spencer, 2004) or sprint exercise (Hamilton et al., 1991) showed higher fatigue during repeated sprints. These findings could be explained by the greater reliance on anaerobic metabolism found in these subjects. Indeed, the present study’s RSA test, composed of 40m all-out sprints, was quite similar in terms of the effort pattern (the mean 40 m sprint time was 5.53 s). Nevertheless, the present study protocol imposed relatively long passive recovery periods between sprints. This allowed a resynthesis of phospocreatine, inducing a high reliance to the anaerobic alactic metabolism and thus quite low post-test lactate values. In the classical RSA testing procedures, the between-sprint rest periods are usually short (25 to 30 s), and thus the effort/recovery ratio is quite different from the test used in the present study.

The protocol applied in the present study took into account the individual loss of performance; therefore, it could explain the differences with previous RSA studies. With a similar number of sprints for all subjects and relatively short recovery periods between sprints, the greater glycolytic energetic contribution within the different sprints has been shown to induce metabolic disorders (higher H^+^ concentration), which is related to glycolytic inhibition (Glaister, 2005) associated with the onset of fatigue as well as the reduction in strength or power output (Sahlin, 1992). With an RSA protocol related to the individual loss of sprint performance, this inhibitory effect could be similar for all players (no differences were found in lactate concentration post-exercise between the most and least powerful players), and therefore other factors such as the ratio of work–recovery should be taken into consideration. This ratio (40-m sprint with 2 min of recovery) was chosen to ensure a certain level of fatigue and decreased performance without compromising the running mechanics as it has been previously demonstrated (Ratel et al., 2006). The 3 min rest between each 10 s sprint allowed the maintenance of a constant running velocity performance, although the peak power output decreased significantly (7%) from the first to the last sprint (Ratel et al., 2006). However, the decreased performance in the 15 × 40 m was statistically significant with 2 min rest periods (Balsom et al., 1992). Consequently, the recovery duration between sprints is a key factor determining performance in these tests, being related to the recovery of the energy substrates employed and the aerobic contribution (Bishop and Spencer, 2004; Dawson et al., 1997; Girard et al., 2011). In 5 × 5-s cycling sprints (Dawson et al., 1997), the recovery of the phosphocreatine stores was higher with longer periods of rest (3 min) compared to shorter periods (10–30 s), whereas the oxygen uptake during the exercise was higher (66%) with 120 s of rest than with 30 s (52%) (Balsom et al., 1992). In this context, it has also been shown that the aerobic contribution also increases with progression of efforts (Chamari et al., 1995).

Moreover, a longer recovery period could induce greater recovery of phosphagen and aerobic contribution than shortened recovery periods, minimizing the appearance of fatigue and reducing the contribution of glycolysis to the total energy output (Glaister, 2005). Thus, the influence of hydrogen ions on both the decrease in intracellular pH and the inhibition of glycolytic energy production caused by the decline of enzyme activity could be reduced (Spriet et al., 1989). The concentration of muscle lactate found after the RSA protocol was (10.1 mmol/l ± 2.1) similar to those previously found in other studies that included the same recovery periods (Balsom et al., 1992), thus confirming this thesis. Therefore, the metabolic interferences mentioned could have a restricting effect on the decrease in RSA performance with longer recovery durations.

The analysis of sprints performed by elite players during the European Champions League and UEFA Cup matches (Di Salvo et al., 2010) revealed that the maximum number of sprints (speed > 25.2 km/h) undertaken during a match ranged according to playing positions from 17.3 ± 8.7 (central defenders) to 35.8 ± 13.4 (wide midfielders). Therefore, this study demonstrated that soccer players might have sufficient recovery time between two maximal sprints (∼5 min in case of central defenders and approx. 2.5 min in case of wide midfielders). Consequently, the metabolic alterations previously discussed would not be decisive in the decreased performance in maximum intensity actions, and other factors like the neuromuscular performance could affect sprint performance.

Unexpectedly, to the best of our knowledge no studies have attempted to examine the relationship between the full squat and loaded jump with RSA in soccer players, whereas sprint performance and power measures have been previously linked (López-Segovia et al., 2011). Several authors have suggested that maximal strength is essential for sprint performance. In this context, different studies with inconclusive results have been presented to assess the relationship between maximal lower body strength (1–3-RM) and sprint performance (Cronin and Hansen, 2005; Harris et al., 2010). The high correlation reported between 1-RM and sprint times (*r* = 0.94–0.71, p ≤ 0.01–0.001) by Wisloff et al. (2004) contrasts with other research that found moderate or non-existent relationships (Cronin and Hansen, 2005). The need to generate strength in a limited time (100–250 ms) at the beginning of a short sprint (Mann and Sprague, 1980) contrasts with the slow movement involved in the lifting of heavy loads. These differences in speed may imply differences in the recruitment of motor units with different activation times for actions, sprinting, and squatting with heavy loads, which would explain the absence of agreement in the previous documented findings. However, the higher velocity that was obtained in the present study by lifting loads near to the relative load that maximized the mechanical power output in full squat (0.8 m/s) could solicit the recruitment of motor units with closer activation times than those required during sprints. It has previously been suggested that explosive movements used for the development of power result more in the high-frequency discharge of the motor units involved compared to slow movements, resulting in different nervous system adaptations (Behm and Sale, 1993). These arguments, along with the risks associated with the determination and use of 1-RM, would suggest that using heavy loads for sprint training in soccer should be reconsidered. Previous findings using the same method to determine the mechanical power output support this suggestion. Indeed, after four months of training and competition (López-Segovia et al., 2010) a significant relationship was reported (*r* = 0.642, p < 0.05) between changes in acceleration and changes in FSL bar speed with loads that were close to the relative load that maximized the mechanical power output during the full squat (65% of 1-RM) and countermovement jumps with external loads as well as volume of strength training performed with these external resistances (*r* = 0.532–0.585, p < 0.05). Coinciding with this study, the results from the present research (Table 3) reported large to very large correlations between sprint times and CMJ_L_ (20–30 kg; *r* = −0.56/−0.79; p ≤ 0.05/0.01) and FS power output with loads close to those that maximized the mechanical power output (65% of 1-RM; *r* = −0.62/−0.78; p ≤ 0.05/0.01) (López-Segovia et al., 2011).

Neuromuscular performance has been shown to be decisive for RSA (Méndez-Villanueva et al., 2008). In the present study, large correlations were observed between both FSMax_MP_·kg^−1^ (*r* = 0.539, *p* ≤ 0.05) and CMJ_20–30–40_·kg^−1^ as well as the RSI (Figure 1). More powerful players were able to sprint more times without loss of performance (Table 2) and they were faster in all sprint measures (Table 2). While neuromuscular activation of the contracting musculature was not measured, previous studies have suggested that sprint decrement is affected by the selective fatigue of fast-twitch fibres because these fibres are fatigued more easily than slow-twitch fibres (Barclay, 1996). This effect occurs in repeated sprint protocols due to, among other factors, greater metabolic alterations (Bishop and Spencer, 2004) as a consequence of shortened recovery periods. Without the inhibitory effect achieved by a longer recovery period, the repeated sprint performance has shown to be related to good mechanical power output of the lower limb.

Therefore, the results obtained could provide valuable information improving soccer players’ performance. Although the relationship does not imply causality, the data obtained suggest that players with higher neuromuscular performance in FS_L_ and CMJ_L_ within the range of the loads that were evaluated could approach the competition with greater assurance of success, given that they demonstrate higher performance in repeated and single sprints.

To assess the differences based on the soccer players’ individual ability and clarify the aspects that are determinant in repeated sprint ability, the use of protocols related to individual performance loss must be utilized more often, because as the present study’s data showed, the differences in lower limb power output can imply different degrees of fatigue with the same number of sprints, and as a result cause different physiological adaptations. These individual differences must be taken into account in prescribing the soccer player’s single and repeated sprint training in order to achieve a high standard of control and understanding of the physiological mechanisms related to the soccer player’s fitness improvement.

## Figures and Tables

**Table 1:** Average results of evaluated variables (means ± SD)

Variables	Mean ± sd
RSI (number of sprints)	7.9 ± 3.2
Time at 10 m (s)	1.91 ± 0.06
Time at 20 m (s)	3.18 ± 0.09
Time at 40 m (s)	5.53 ± 0.18
Lactate_Pre_	3.12 ± 1.13
Lactate_Post_	10.1 ± 2.09
CMJ_20–30–40_ (w·kg^−1^)	14.84 ± 2.53
Full squat maximal mean power (w)	558 ± 97
Full squat maximal mean power/kg (w·kg^−1^)	7.44 ± 1.5
Full squat maximal mean power load (kg)	65.9 ± 10.6

RSI = repeated sprint index, number of 40 m sprints executed by a player until there was a 3% decrease in the best 40 m performance; Time at 10 m = best 10 m sprint; Time at 20 m = best 20 m sprint; Time at 40 m = best 40 m sprint; Lactate_Pre_ = blood lactate concentration before warm-up; Lactate_Post_ = blood lactate concentration at the end of repeated sprint protocol; CMJ_20–30–40_ (w·kg^−1^) = sum of peak power in CMJ with 20, 30, and 40kg per kg of body mass; Full squat maximal mean power (w) = maximal mean power during the concentric phase obtained in progressive test with external load; Full squat maximal mean power/kg (w·kg^−1^) = maximal mean power during the concentric phase obtained in progressive test with external load per kg of body mass; Full squat maximal mean power load (kg) = load that maximized the mechanical power output in FS.

**Table 2:** Comparison between the most and least powerful group of players (FSMax_MP_ and CMJ_20–30–40_) related to body mass (means ± SD)

	FS_L_		CMJ_L_	

Variables	Most FSMax_MP_ (w·kg^−1^) *N* = 9	Least FSMax_MP_ (w·kg^−1^) *N* = 9	Most PPCMJ_20–30–40_(w·kg^−1^) *N* = 9	Least PPCMJ_20–30–40_ (w·kg^−1^) *N* = 9
RSI	9.1 ± 4.2	6.5 ± 1.6^*^	8.9 ± 4	6.6 ± 2.2
Time 10 m(s)	1.87 ± 0.03	1.93 ± 0.08^**^	1.88 ± 0.05	1.92 ± 0.07
Time 20 m(s)	3.13 ± 0.05	3.23 ± 0.11	3.15 ± 0.07	3.2 ± 0.11
Time 40 m(s)	5.46 ± 0.12	5.63 ± 0.21	5.47 ± 0.13	5.59 ± 0.23
Lactate_Pre_	2.93 ± 0.74	3.61 ± 1.4^*^	3.03 ± 0.96	3.37 ± 1.4
Lactate_Post_	10.41 ± 2.6	9.65 ± 1.7	9.73 ± 2.57	10.26 ± 1.9

Intergroup analysis= ^*^p ≤ 0.05;

** p ≤ 0.01. FSL = progressive test with external load in full squat;

CMJL = progressive test with external load in countermovement jump. Most/Least FSMaxMP = player’s data with most/least maximal mean power obtained in progressive test with external load in full squat related to body mass; Most/least PPCMJ20–30–40 = player’s data with most/least sum of peak power output with 20 kg, 30 kg, and 40 kg in progressive test in coutermovement jump relative to body mass; RSI = repeated sprint index, number of 40 m sprints executed by the player until there was a 3% decrease in the best 40 m performance; Time 10 m = best 10 m sprint; Time 20 m = best 20 m sprint; Time 40 m = best 40 m sprint; LactatePre = blood lactate concentration before warm-up; LactatePost = blood lactate concentration at the end of repeated sprint protocol.

**Table 3:** Correlations between muscle power output variables and sprint performance

Variables	FS_MP_								PPCMJ_L_			

	Max_MP_	Max_MP_·kg^−1^	External load (kg)						PP_20–30–40_·kg^−1^	PP_20_	PP_30_	PP_40_
			**20**	**30**	**40**	**50**	**60**	**70**				
RSI	.411	.539^*^	−.116	.055	.215	.278	.221	.341	.591^*^	.520^*^	.640^**^	.500^*^
Time 10 m (s)	−.573^*^	−.501^*^	−.194	−.411	−.566^*^	−.434	−.539^*^	−.613^*^	−.547^*^	−.453	−.482	−.619^*^
Time 20 m (s)	−.704^**^	−.691^*^	−.307	−.478	−.724^**^	−.587^*^	−.727^**^	−.725^**^	−.640^**^	−.548^*^	−.557^*^	−.663^**^
Time 40 m (s)	−.627^**^	−.660^**^	−.318	−.428	−.621^**^	−.492^*^	−.682^**^	−.687^**^	.568^*^	−.534^*^	−.480	−.559^*^

FS_MP_ = the best datum for mean power output during the concentric phase in the repetitions done with each external load; PPCMJ_L_ = the best data for peak power during the concentric phase of the repetitions done with each external load; FSMax_MP_ = power output registered with the load that maximized the mechanical power output in FS_L_ test; FSMax_MP_·kg^−^^1^ = power output registered with the load that maximized the mechanical power output in FS_L_ test per kg of body mass; PPCMJ_L_ = the best data for peak power during the concentric phase of the repetitions done with each external load; PPCMJ_20–30–40_·kg^−^^1^ = the sum of the maximum peak power output with 20 kg, 30 kg, and 40 kg in CMJ_L_ test per kg of body mass; RSI = repeated sprint index, number of 40 m sprints executed by the player until there was a 3% decrease in the best 40 m sprint; Time 10 m = best 10 m sprint; Time 20 m = best 20 m sprint; Time 40 m = best 40 m sprint. (

* p ≤ 0.05;

** p ≤ 0.01).
